# Optimal Design of Multi-Scale Fibre-Reinforced Cement-Matrix Composites Based on an Orthogonal Experimental Design

**DOI:** 10.3390/polym15132898

**Published:** 2023-06-30

**Authors:** Kaixin Qiu, Song Chen, Chen Wang, Bowei Yang, Jiuhong Jiang

**Affiliations:** College of Civil Engineering, Architecture and Environment, Hubei University of Technology, Wuhan 430068, China; 102110917@hbut.edu.cn (K.Q.); 102010881@hbut.edu.cn (S.C.); 102010862@hbut.edu.cn (C.W.); 102110969@hbut.edu.cn (B.Y.)

**Keywords:** multi-scale fibres, orthogonal experiments, grey correlation analysis, MSFRCC, fly ash

## Abstract

Cement-matrix composite are typical multi-scale composite materials, the failure process has the characteristics of gradual, multi-scale and multi-stage damage. In order to delay the multi-stage damage process of cement-matrix composites, the defects of different scales are suppressed by using different scales of fibres and fly ash (FA), and the overall performance of cement-matrix composites is improved, a new multi-scale fibre-reinforced cement-based composite composed of millimetre-scale polyvinyl alcohol fibre (PVA), micron-scale calcium carbonate whisker (CW), and nano-scale carbon nanotubes (CNTs) was designed in this study. The compressive strength, flexural strength, splitting tensile strength, and chloride ion permeability coefficient were used as assessment indices by the orthogonal test design. The impacts of the three fibre scales and fly ash on each individual index were examined, and the overall performance of the multi-scale fibre-reinforced cementitious materials (MSFRCC) was then optimized using grey correlation analysis. The optimized mix ratio for overall performance was PVA: 1.5%, CW: 2%, CNTs: 0.1%, FA: 40%. Compared with the optimal results for each group, the compressive strength of the final optimized MSFRCC group decreased by 8.9%, the flexural strength increased by 28.4%, the splitting tensile strength increased by 10%, and the chloride ion permeability coefficient decreased by 5.7%. The results show that the compressive performance and resistance to chloride ion penetration of the optimized group are slightly worse than those of the optimal group in the orthogonal test, but its flexural performance and splitting tensile performance are significantly improved.

## 1. Introduction

Cement-matrix composite are widely used in civil engineering because of their high compressive strength, wide distribution of raw materials, easy access to available materials, and low price. Cement-matrix composite do, however, have some important downsides, such as low tensile strength and crack susceptibility, which might reduce the durability and service life of projects [1]. Many academics have suggested using fibres to improve the performance of cement-matrix composites to meet the demands of modern buildings and overcome the disadvantages of cement-matrix composites mentioned above [2,3,4]. Adding fibre to concrete can improve the safety, performance, service life, and durability of concrete structures [5]. Cement-matrix composite have obvious multi-level structural characteristics, and their structures contain four levels: nanoscale, microscopic, fine-scale, and macroscopic [6]. The damage process of cement-matrix composites is also characterized by progressive, multi-scale, and multi-stage damage [7,8]. When external loads are applied, microscopic fissures initiate within the cement-matrix composite foundation, which evolve into fine cracks and then develop into macroscopic cracks as the load continues to increase, eventually leading to the destruction of the cement-matrix composite [3,9,10,11,12,13] As a result, it is difficult for single-scale fibres to adapt to cement-matrix composite multi-scale structures and multi-stage damage [14,15,16].

Polyvinyl alcohol fibres (PVA) have been used as reinforcing materials for cement-matrix composites because of their high tensile strength and modulus of elasticity [17,18,19]. According to Loh et al. [20], PVA fibres significantly increased tensile and flexural strengths but had little impact on compressive strength. Pakravan et al. [21] found that the flexural strength and flexural modulus of cement-matrix composites increased significantly with increasing PVA fibre content; however, the toughness index decreased with increasing fibre admixture at higher fibre admixture levels. According to Mostofinejad et al. [22], mixing PVA and polypropylene (PP) fibres can significantly increase the flexural strength, toughness, and energy absorption of cement-matrix composites. After the PVA fibre content was increased, it significantly increased the ability of cement-matrix composites to absorb energy, according to research by Atahan et al. [23] into the flexural characteristics and impact resistance of such materials. According to Nam et al. [24], the addition of PVA fibres significantly increased the frost resistance of cement-matrix composites.

Calcium carbonate whiskers (CW), as micron-sized fibre materials with high strength, high modulus of elasticity, and high aspect ratio, can retard the expansion and extension of fine cracks in cement-matrix composites [25,26]. Studies have shown that microfibres shorter than 0.1 mm are more evenly distributed in concrete, resulting in a higher bulk density of the cement matrix [27]. After Cao et al. [28] and Li et al. [29] created new hybrid cement-matrix composites reinforced with steel, PVA, and CW, the results revealed that the interactions between the fibres on various scales had a positive impact on the mechanical properties of the cement-matrix composites. Li et al. [30] further investigated the mechanical properties and microstructure of such new hybrid fibre-reinforced cement-matrix composites at high temperatures, and the structures showed that the multiscale hybrid fibre system can mitigate the deterioration of the mechanical properties of cement-matrix composites at high temperatures. Yuan et al. [31] used CW fibres to partially replace PVA fibres to investigate the flexural properties of cement-matrix composites with levels of PVA fibre and CW ratios, and the results showed that the flexural strength, ultimate flexural deflection, and flexural toughness of cement-matrix composites were effectively improved with increasing amounts of CW admixture.

Carbon nanotubes (CNTs), as a new type of nanomaterial, can fill the cement microstructure and reduce the porosity of cement-matrix composites by filling the pores between hydration products, thus achieving enhanced mechanical properties and durability of cement-matrix composites [32,33,34]. Gao et al. [35] found that CNTs can not only promote cement hydration reactions to form more hydration products in the interfacial transition zone but also inhibit the initiation of microscopic cracks and improve the permeability of concrete. Mohsen et al. [36] found that CNTs are effective in reducing the permeability of concrete when they are incorporated into concrete. Irshidat et al. [37] found that CNTs were able to enhance the residual compressive and flexural strength of PP fibre-reinforced cement-matrix composites at high temperatures, and SEM images showed that CNTs filled the pores inside the cement-matrix composites and effectively retarded the sprouting of cracks. Lee et al. [38] found that the resistance of cement-matrix composites to sulfuric acid corrosion was significantly improved when CNTs were incorporated, and CNTs could effectively prevent cement hydration products from coming into contact with sulfuric acid solutions. Jazaei et al. [39] found that CNTs can enhance energy dissipation by cement-matrix composites, and composites doped with 0.4 wt% heterogeneous CNTs had the highest energy dissipation capacity, while CNTs could improve the damage morphology by reducing crack extension.

Fly ash (FA) is widely used as a supplementary cement-matrix composite in the construction industry [40]. The use of FA as a partial replacement of cement in concrete is generally limited to 15% to 20% of the total cement-matrix composite mass [41]. However, the yearly increase in FA production has led to extensive research on composites more highly doped with FA as well. Arezounnandi et al. [42] found that highly doped FA concrete beams had high flexural strength. Liu et al. [43] found that high volumes of FA not only refined the pore size but also improved the pore size distribution of the cement matrix, which greatly contributed to the development of the microstructure as well as the strength and durability of the concrete. Wongkeo et al. [44] found that the CaO content in FA had a significant effect on the hydration reaction of cement and that high dosing of FA helped to increase the early compressive strength of concrete. Wang et al. [45] found that FA mainly acted as a diluent and volcanic ash reactant, where the dilution effect referred to FA replacing part of the cement, increasing the water-to-cement ratio and improving cement hydration.

The multi-scale fibre reinforced cementitious composites proposed by Montero-Chacon et al. [46] are improved by changing the length of macro-fibres and dividing the fibres above 1 mm into three different lengths and diameters. The multi-scale fibre reinforced cementitious composites studied by Zhang et al. [47] added steel fibre, polyvinyl alcohol fibre, and calcium carbonate whiskers, and tested the fibre synergy in terms of compressive and flexural properties. The multi-scale fibre-reinforced cement-matrix composites studied by Masud [48] and others use macro, micron, and nano-scale fibres to test their mechanical properties and significantly improve their strength. However, at present, the above research lacks the research on fibres of different scales, and also does not optimize the design of their comprehensive properties. In order to improve the multi-scale structure and multi-level damage process of cement-matrix composites, millimetre-scale PVA fibre, micron-scale CW fibre, and nano-scale CNTs fibre are used to improve the defects of different sizes in this paper, and a new type of MSFRCC is designed to improve the comprehensive performance of cement-matrix composites. Liu et al. [49] used an orthogonal test design method to reduce the number of tests and the overall cost; the orthogonal test method is widely used to optimize design parameters. This study was based on an orthogonal experimental design. The effects of three different scales of fibre and FA doping on the individual mechanical properties or durability of MSFRCC were investigated with PVA fibre doping, CW doping, CNTs doping, and FA doping as the influencing factors, and the comprehensive performance of MSFRCC was optimized by using grey correlation analysis.

## 2. Materials and Methods

### 2.1. Test Materials

The materials used in the test were mainly as follows: the cement was P·O 42.5 grade ordinary silicate cement, and the FA was first-grade fly ash. The aggregate was IOS standard sand, and the particle size control index of the IOS standard sand is shown in Table 1. The fibres used were millimetre-scale PVA fibres, micron-scale CW, and nanoscale CNTs, and the index properties of each fibre are shown in Table 2. The water-reducing agent was a polycarboxylic acid water-reducing agent with 25% water reduction. The defoamer was tributyl phosphate. The test water was ordinary tap water.

### 2.2. Matching Ratio Design

In this test, the water–cement ratio was 0.3, and the sand–cement ratio was 0.5. The volume fraction of PVA fibre (A: V_PVA_), the volume dose of CW (B: V_CW_), the mass dose of CNTs (C: M_CNTs_), and the dose of FA (D: FA) were selected, the total cement substitution was the influencing factor, and a four-factor, three-level orthogonal test, a total of 9 groups of mix ratio L9 (34) was designed. The key to orthogonal experimental design is the arrangement of experimental factors. Usually, without considering the interaction, you can freely arrange each factor in each column of the orthogonal table, as long as you do not arrange two factors in the same column. However, when considering the interaction, it will be subject to certain restrictions. If arranged arbitrarily, it will lead to the interaction effect mixed with other effects. Therefore, the interaction was not considered in this experiment. Factors and levels are shown in Table 3, and specific fits are shown in Table 4.

### 2.3. Specimen Preparation and Test Methods

#### 2.3.1. Dispersion of CNTs

**Figure 1:**
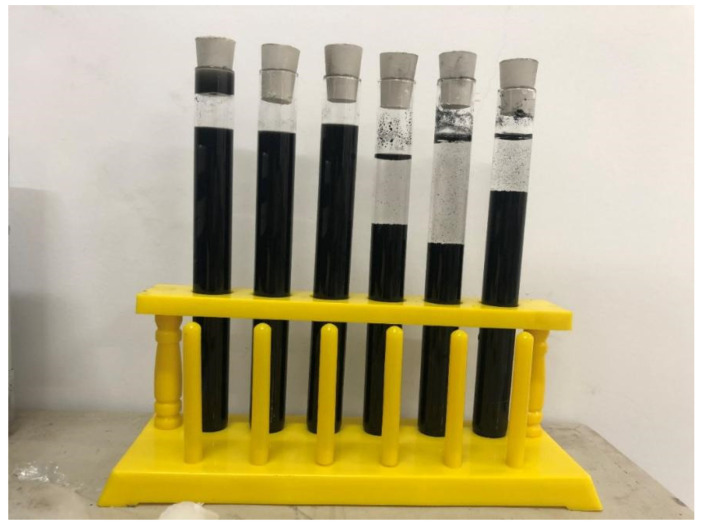
Dispersion-treated CNTs solution and undispersion-treated CNTs solution.

Due to their small size, large specific surface area, and high aspect ratio, CNTs are prone to entanglement or agglomeration in aqueous solutions, resulting in difficulty in dispersing CNTs in cementitious composites [34]. Thus, dispersion treatment of CNTs is required before adding CNTs. In this experiment, the ultrasonic method [50] was chosen to combine X with the surfactant method [51], polyvinylpyrrolidone (PVP) was used as the surfactant, and the mass ratio of CNTs to PVA was 1:2. The CNTs and dispersant were dispersed in combination with ultrasonic treatment; the concentration of dispersant was 1%. The specific process was as follows:

(1) First, the dispersant was slowly added to distilled water, and the solution was placed into a heat-collecting magnetic stirrer for 2–3 min. (2) CNTs were slowly added, and magnetic stirring was continued for 2–3 min. (3) The stirred CNTs dispersion was placed into an ultrasonic cleaner for ultrasonic dispersion for 45 min, and the dispersion was removed and placed in ice water for 5 min every 15 min for cooling treatment. (4) Finally, the beaker was covered with a film to prevent water evaporation. The dispersed CNTs solution and the undispersed CNTs solution are shown in Figure 1. The undispersed CNTs solution exhibited obvious delamination and sedimentation, while the dispersed CNTs solution was well stabilized, and no obvious sedimentation occurred.

#### 2.3.2. Preparation of Multi-Scale Fibre-Reinforced Concrete

The preparation process was as follows: (1) CW was added together with cement, FA, and other cementitious materials into the mixer and dry stirred for 2 min. (2) Standard sand was poured in, and the mixture was dry stirred for 2 min. (3) CNTs were dispersed uniformly with water and a water-reducing agent to form a mixture. (4) The mixture was poured into the mixer and stirred for 3 min. (5) PVA fibre was added evenly into the mixer and stirred for 2 min. (6) Defoamer was added and stirred for 1 min. (7) The finished concrete was poured into the mould, and the mould was placed on the vibrating table to vibrate until it became dense. The specimens were demoulded 1 d after moulding and then placed in a standard curing room (temperature 20 ± 2 °C, humidity 95% or more) for 28 d.

#### 2.3.3. Basic Mechanical Properties and Durability Testing

The compressive strength test was performed as in JGJ/T70-2009 [52]. In this method, a cube specimen with a size of 70.7 mm is used, and the loading speed is 0.5 kN/s.

The flexural test was GB/T 17671-2021 [53]. This method uses prismatic specimens with dimensions of 40 mm × 40 mm × 160 mm, and the loading speed is 0.05 kN/s.

The splitting tensile test was GB/T50081-2002 [54]. The test was performed with a cubic specimen with a size of 100 mm, and the loading speed was 0.05 MPa/s.

The resistance to chloride ion penetration test was designed according to GB/T 50082-2009 [55]. This test uses the rapid chloride migration coefficient method (RCM method), and the RCM test uses a cylindrical specimen of size φ 100 mm × 50 mm. The RCM test procedure was as follows: First, the test block was treated with vacuum saturation, and then an electric field was applied at both ends of the test piece. Next, 300 mL of NaOH solution with a concentration of 0.3 mol/L was injected into the anode test tank, and 12 L of NaCl solution with a mass fraction of 10% was injected into the cathode test tank to accelerate the transport of chloride ions inside the cementitious material. After the power was turned on, the voltage was adjusted to 30 ± 0.2 V, the initial current through each specimen was recorded, the voltage to be applied for the subsequent test was selected according to the initial current. The new current was recorded, and finally the time that the test should last was determined according to the new current. After the test, the specimen was split into two halves along the axial direction, and 0.1 mol/L AgNO_3_ solution was sprayed on its split section. After waiting for 15 min, the split section of the specimen was divided into 10 equal parts, and then the penetration contour line was traced. Finally, the distance *X_i_* of the colour change at dividing line from the bottom surface of the specimen was measured according to the observed obvious colour change. The chloride ion penetration depth *X_d_* was the average value of *X_i_*.

The non-stationary chloride ion migration coefficient was calculated as shown in Equation (1):

(1) $$D_{RCM}=\frac{0.0239\times (273+T)L}{(U-2)t}(X_{d}-0.0238\sqrt{\frac{(273+T)LX_{d}}{U-2}})$$

in the formula: *D*_RCM_—Non-stationary chloride ion mobility coefficient (m^2^/s);

*U*—Absolute value of voltage (V);

*T*—Average of the initial and ending temperatures of the anode solution (°C).

**Figure 2:**
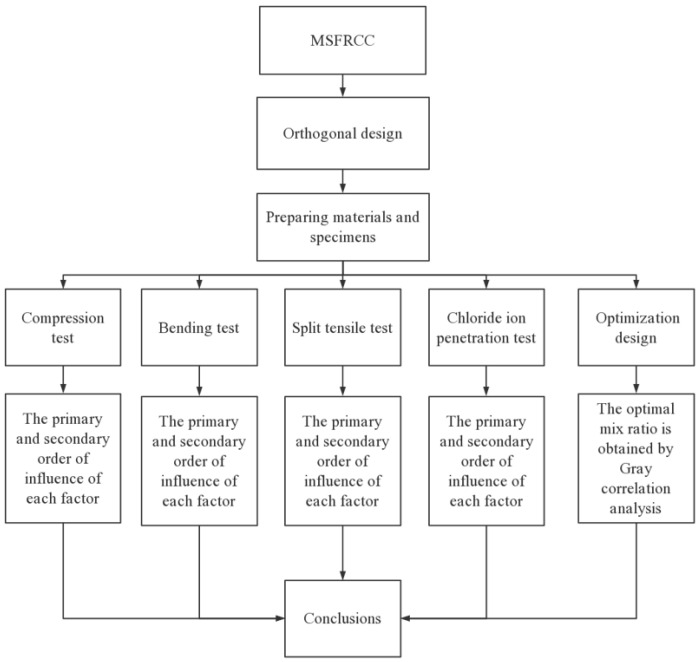
Experimental design flow chart.

The experimental process is shown in Figure 2:

## 3. Results and Discussion

Two processing methods were used for orthogonal test data, range analysis and analysis of variance (ANOVA). Range analysis refers to the calculation of the range of orthogonal test data. If the sum of the data corresponding to the same level under the same factor is K, then the mean value of the data corresponding to the level is ki, and the range of the same factor at different levels is R. The variance analysis is divided into the following steps. First, the total sum of squared deviations, the sum of squared deviations corresponding to each factor, and the sum of squared deviations caused by errors are calculated. On this basis, the F test is carried out, and finally, the significance of the influence of each factor on the target performance is evaluated, and the contribution is calculated. Since the orthogonal table in the orthogonal test design of this chapter is full of factors, the error must be provided by repeated test data. In this experiment, two groups were tested each time, and each group contained three specimens.

### 3.1. Compressive Strength

**Figure 3:**
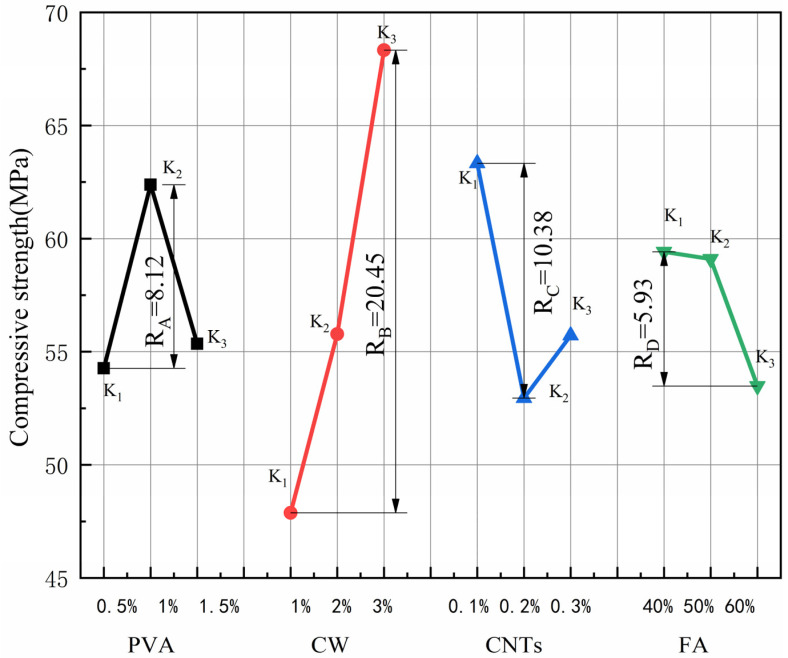
Effect of various factors on the compressive strength.

The results for compressive strength and extreme difference analysis are shown in Table 5, and the variation of the extreme difference R-value of each factor is shown in Figure 3. According to standard deviation less than 1%, the test dispersion is not large, so the test data are reliable. Since the compressive strength is the efficiency index, a larger K value represents better compressive performance. By comparing the R-values between the factors, it can be concluded that the main order of influence of the four factors on the compressive strength is R_B_ > R_C_ > R_A_ > R_D_. CW has the greatest effect on compressive strength. In the range of CW dosing levels, the compressive strength increases with increasing CW dosing, and the best compressive performance of the specimen is obtained when the CW dosing is 3%. The effect of CNTs on compressive strength is second only to CW. When the CNTs dose is 0.1%, the specimen has the best compressive performance, and when the CNTs dose exceeds 0.1%, the compressive strength tends to decrease. The effect of PVA fibres on the compressive strength is relatively small when the PVA fibre doping amount reaches 1%, and the best effect of compressive strength enhancement occurs when the PVA fibre doping amount exceeds 1%. When the PVA fibre doping amount reaches 1.5%, the compressive strength tends to decline, and the K value is similar to that when the fibre doping amount is 0.5%. In contrast, the effect of the FA admixture on the compressive strength is the smallest, the compressive strength is the largest when the FA admixture reaches 40%, and the compressive strength gradually decreases with the increase in the FA admixture in the range of the FA admixture level.

The addition of CW and CNTs filled the micro-nano pores of the cement-based composites, refined the pores, and made the interior of the matrix dense, thereby improving the compressive strength. However, because the size and content of CNTs are much smaller than that of CW, the effect of CNTs on compressive strength is less than that of CW. PVA and FA did not have a significant effect on the fine pores of the sample, so CW had the greatest effect on the compressive strength. At the same time, Cao et al. [28] showed that CW has a significant positive effect on the compressive strength of cement-based composites. Cao et al. [47] showed that the maximum compressive strength of MSFRCC with only PVA and CW was 59.2 MPa, and the maximum compressive strength of MSFRCC designed by orthogonal test was 81.2 MPa, which significantly improved its compressive strength.

It is obvious from Figure 3 that when the PVA fibre admixture is 1%, the CW admixture is 3%, the CNTs admixture reaches 0.1% and the FA admixture is 40%, the compressive strength of MSFRCC reaches the maximum, and the matching ratio with the optimal effect of compressive strength enhancement is A2B3C1D1 (PVA: 1%, CW: 3%, CNTs: 0.1%, FA: 40%).

**Figure 4:**
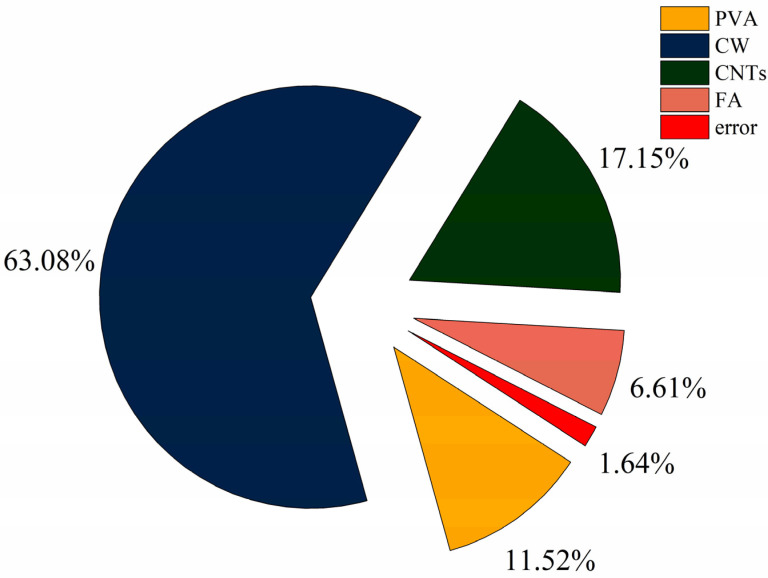
Contribution rate of each influencing factor to compressive strength.

The results of the ANOVA for compressive strength are shown in Table 6. The distribution of the contribution is shown in Figure 4. According to Table 6, the four factors are ranked in order of significance: B(V_CW_) > C(M_CNTs_) > A(V_PVA_) > D(FA). In Figure 4, the degree of influence of CW on compressive strength is much greater than the other three factors, reaching 63.08%, the degree of influence of PVA fibres and CNTs on compressive strength is equal, 11.52%, and 17.15%, respectively, and the degree of influence of FA on compressive strength is 6.61%, the lowest among all the factors. For improving the compressive strength of MSFRCC, the incorporation of CW is the most effective.

### 3.2. Flexural Strength

**Figure 5:**
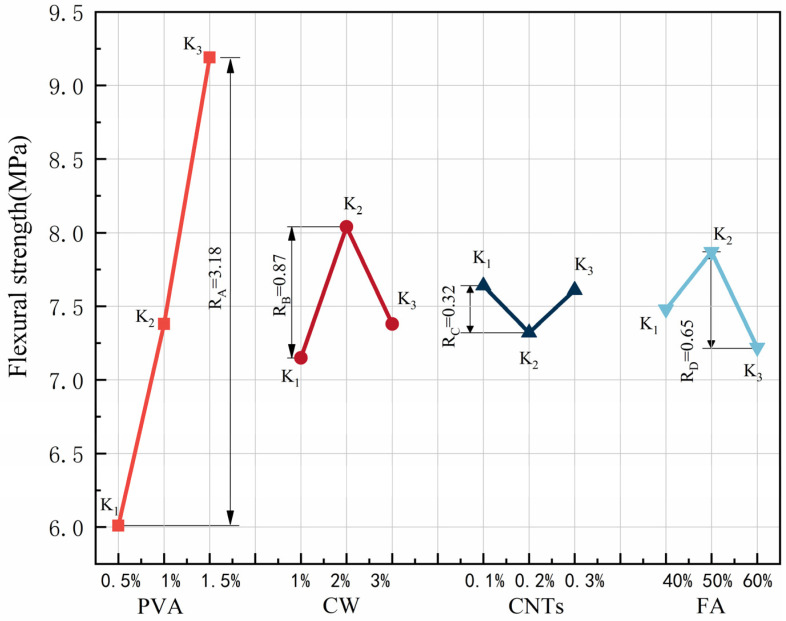
Effect of various factors on the flexural strength.

The results for flexural strength and extreme difference analysis are shown in Table 7, and the variation of the extreme difference R-value of each factor is shown in Figure 5. According to standard deviation less than 1%, the test dispersion is not large, so the test data are reliable. Since the flexural strength is a beneficial index, a larger K value represents better flexural performance. By comparing the R-values among the factors, we found that the main order of the influence of the four factors on flexural strength was R_A_ > R_B_ > R_D_ > R_C_. PVA fibre had the greatest effect on flexural strength, far exceeding the other three factors. In the range of PVA fibre doping levels, the flexural strength increased with increasing PVA fibre doping, and the best flexural performance of the specimens was achieved when the PVA fibre doping reached 1.5%. The effect of CW on flexural strength was second only to that of PVA fibre. When the CW dose reached 2%, the specimen had the best flexural performance, and when the CW dose exceeded 2%, the flexural strength started to decrease. The effect of FA on flexural strength was relatively small. When the FA dosing reached 50%, the flexural strength of the specimen was the best, and when the FA dosing exceeded 50%, the flexural strength had a tendency to decrease. When the FA dosing reached 60%, the flexural strength of the specimen was worse than when the FA dosing was 40%. In contrast, CNTs had the least effect on flexural strength, and the specimens showed the best flexural performance when the CNTs dose was 0.1% and the worst flexural performance when the CNTs dose reached 0.2%.

PVA fibre has high elastic modulus, which can significantly inhibit its development of macro cracks. The addition of CW delays the development of cracks at the micron level. The addition of FA can enhance the toughness of cement-based composites. CNTs have little effect on inhibiting the development of macro cracks because of their small content, so the effect is minimal. CW, CNTs, and FA are used to improve the microstructure, so PVA fibre has the greatest influence on the flexural strength. At the same time, Pakravan et al. [21] found that the addition of PVA fibres can significantly improve the flexural strength of cement-based composites. Shokrieh et al. [56] showed that the maximum flexural strength of single-doped PVA was less than 3.5 MPa, and the maximum compressive strength of MSFRCC designed by orthogonal test was 9.5 MPa, which significantly improved its flexural strength.

In Figure 5, the flexural strength of MSFRCC reached the maximum when the PVA fibre doping was 1.5%, CW doping was 2%, CNTs doping reached 0.1%, and FA doping was 50%, and the optimal ratio for flexural strength enhancement was A3B2C1D2 (PVA: 1.5%, CW: 2%, CNTs: 0.1%, FA: 50%).

**Figure 6:**
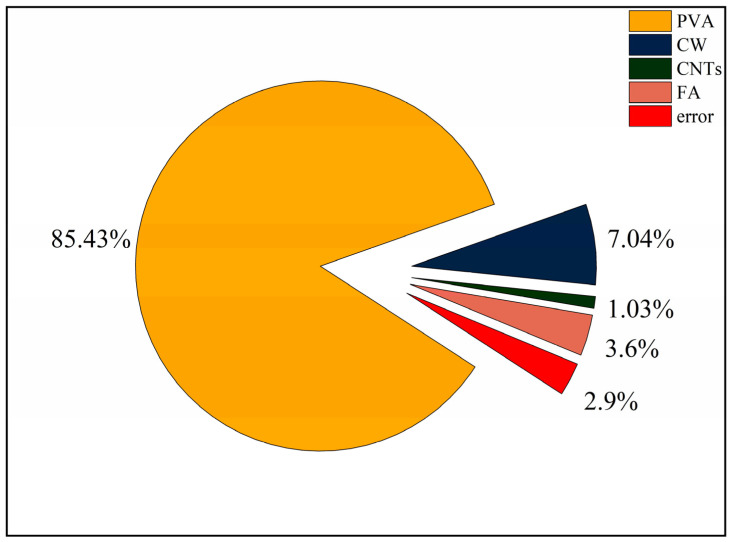
Contribution rate of each influencing factor to flexural strength.

The results of ANOVA for flexural strength are shown in Table 8. The contribution distribution is shown in Figure 6. According to Table 8, it can be learned that the four factors are ranked in order of significance: A(V_PVA_) > B(V_CW_) > D(FA) > C(M_CNTs_). It can be seen from Figure 6 that the degree of influence of PVA fibre on the compressive strength is much greater than the other three factors, with a contribution rate of 85.43%, which is the most important factor affecting the flexural properties. In addition, CW, CNTs, and FA have much less influence on flexural strength than PVA fibres, with their contributions of 7.04%, 1.03%, and 3.6%, respectively, and are not major factors in the design of the ratio with flexural properties as the performance target.

### 3.3. Splitting Tensile Strength

**Figure 7:**
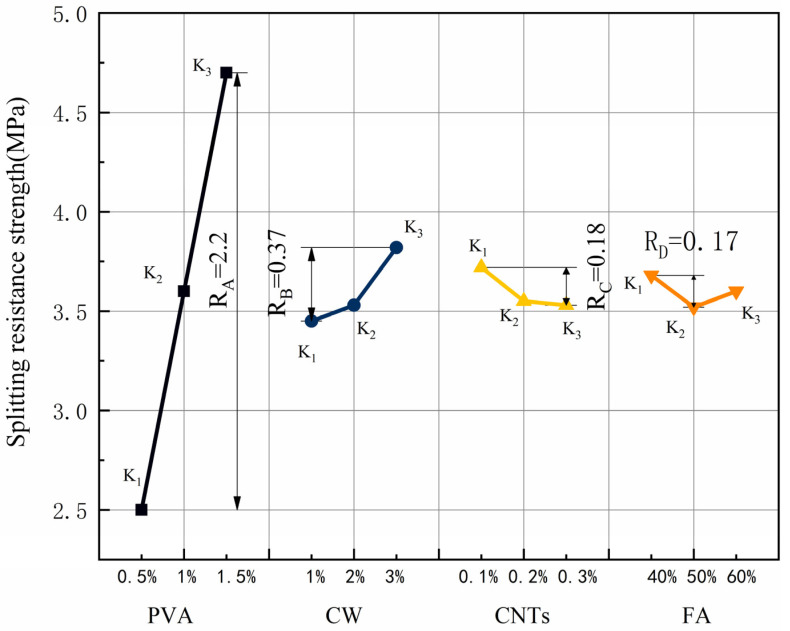
Effect of various factors on the splitting tensile strength.

The splitting tensile strength and the results of the extreme difference analysis are shown in Table 9, and the variation of the extreme difference R-value of each factor is shown in Figure 7. According to standard deviation less than 1%, the test dispersion is not large, so the test data are reliable. Since the splitting tensile strength is the index of benefit, a larger K value represents better folding resistance. Comparing the R-values between the factors showed that the main order of influence of the four factors on the splitting tensile strength was R_A_ > R_B_ > R_C_ > R_D_. The effect of PVA fibre on the splitting tensile strength was the greatest, with R_A_ reaching 2.2, which far exceeded the other three factors. In the range of PVA fibre doping levels, the splitting tensile strength increased with increasing PVA fibre doping, and the best splitting tensile performance of the specimens was obtained when the PVA fibre doping reached 1.5%. The effect of CW on splitting tensile strength was second only to that of PVA fibre. In the range of CW doping levels, the splitting tensile strength increased with increasing CW doping, and the best splitting tensile performance of the specimens was achieved when the CW doping reached 3%. The effect of CNTs on the splitting tensile properties was relatively small, and the splitting tensile strength is maximum when the CNTs dose was 0.1%. When the CNTs dose exceeded 0.1%, the splitting tensile strength started to have a decreasing trend, and the splitting tensile strength was minimum when the CNTs dose reached 0.3%. The effect of FA on the splitting tensile strength was the smallest among the four factors, and the specimen had the best splitting tensile performance when the FA dose was 40%, and the specimen had the worst splitting tensile performance when the FA dose reached 50%, but the change in FA dose had the least effect on the performance, and the R_D_ was only 0.17.

PVA as a macro fibre can also improve the splitting tensile strength of the specimen. The addition of CW and CNTs refines the pores, and PVA fibres together limit the development of macro cracks and micro cracks. In contrast, FA has the least effect on splitting tensile strength. Zhang et al. [57] found that PVA can improve the splitting tensile strength, and PVA fibre limits the development of macro cracks and micro cracks in the splitting situation. Antroula et al.’s [58] research showed that the maximum splitting tensile strength of single-doped PVA was 4.27 MPa, and the maximum splitting tensile strength of MSFRCC designed by this orthogonal test was 5.0 MPa, which only slightly increased its splitting tensile strength. This is because the size of CW and CNTs is small, which is used to delay the development of small cracks, but the inhibition effect on macroscopic cracks is not obvious.

It is obvious from Figure 6 that the splitting tensile strength of MSFRCC will reach the maximum when the PVA fibre dose is 1.5%, the CW dose is 3%, the CNTs dose reaches 0.1%, and the FA dose is 40%, and the optimum ratio for splitting tensile strength enhancement is A3B3C1D1 (PVA: 1.5%, CW: 2%, CNTs: 0.1%, FA: 40%).

**Figure 8:**
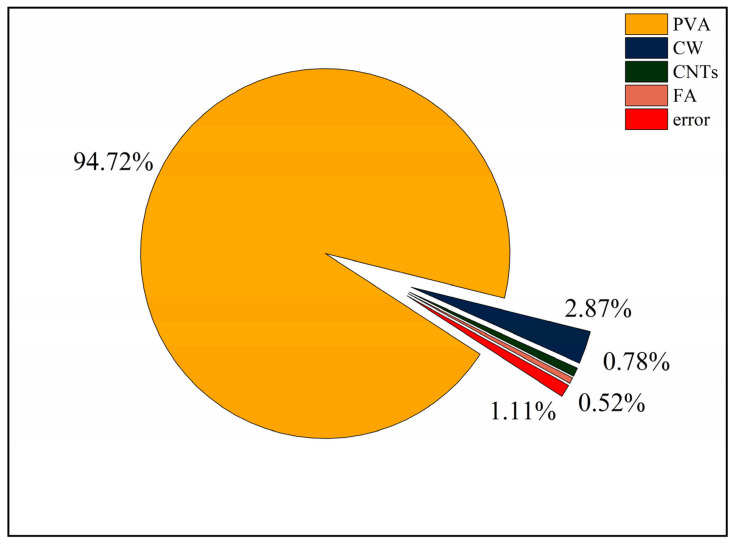
Contribution rate of each influencing factor to splitting tensile strength.

The splitting tensile strength ANOVA results are shown in Table 10. The contribution distribution is shown in Figure 8. According to Table 10, the four factors were ranked in order of significance: A(V_PVA_) > B(V_CW_) > C(M_CNTs_) > D(FA). From Figure 8, the degree of influence of PVA fibre on splitting tensile strength was much greater than the other three factors, with a contribution of 94.72%, which was the most important factor affecting the splitting tensile performance. The degrees of influence of CW, CNTs, and FA on splitting tensile properties were much smaller than that of PVA fibres, whose contributions were 2.87%, 0.78%, and 0.52%, respectively, and it was not a major factor in the design of the ratio with splitting tensile properties as the performance target.

### 3.4. Resistance to Chloride Ion Penetration

**Figure 9:**
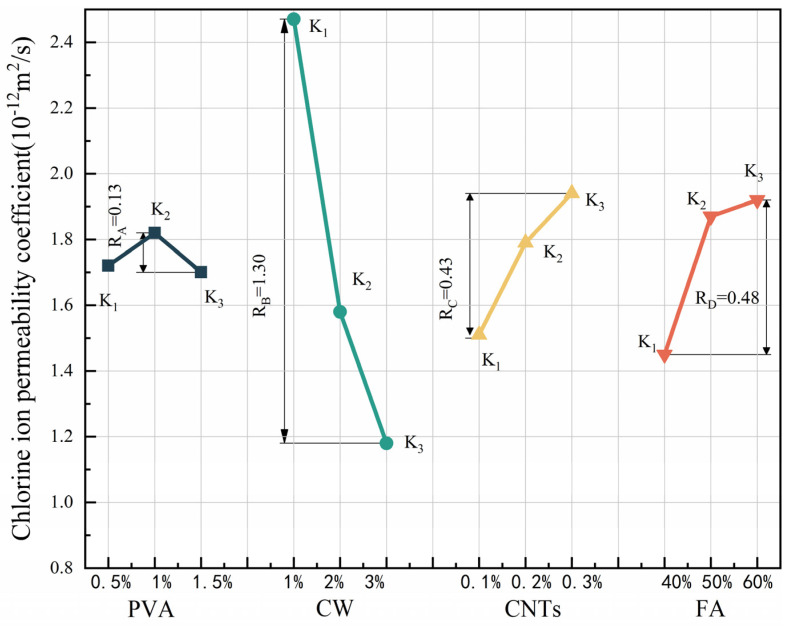
Effect of various factors on the chloride ion permeability coefficient.

The results for the chloride ion permeability coefficient and polar difference analysis are shown in Table 11, and the variation pattern of the R-value of polar difference for each factor is shown in Figure 9. According to standard deviation less than 1%, the test dispersion is not large, so the test data are reliable. Since the chloride ion permeation coefficient is a negative indicator, a smaller K value represents better resistance to chloride ion permeation. Comparing the R-values between the factors showed that the main order of influence of the four factors on flexural strength was R_B_ > R_D_ > R_C_ > R_A_. CW had the greatest effect on the chloride ion permeation resistance and was the most important factor affecting the chloride ion permeation resistance of the specimen. In the range of CW doping levels, the chloride ion permeation coefficient decreased with increasing CW doping; the greater the CW doping was, the better the resistance to chloride ion permeation, and when the CW doping reached 3%, the specimen had the best resistance to chloride ion permeation. The effect of FA on the chloride ion permeation resistance was second only to CW. The chloride ion permeation coefficient increased with increasing FA amount, and in the horizontal range, the higher the FA doping amount was, the worse the chloride ion permeation resistance. When the FA doping amount was 40%, the specimen had the best resistance to chlorine ion permeation performance. The degree and relationship of the effect of CNTs on the resistance to chloride ion permeation were similar to those of FA. In the horizontal range, the larger the CNTs dose was, the worse the resistance to chloride ion permeation. The best resistance to chloride ion permeation of the specimens was achieved when the CNTs dose was 0.1%. In contrast, PVA fibre had the least effect on the resistance to chloride ion permeation. When the PVA fibre doping amount reached 1.5%, the specimen had the best resistance to chloride ion permeation, and when the PVA fibre doping amount was 1%, the specimen had the worst antichlorine ion permeation performance.

CW has the greatest influence on chloride ion permeability. This is because the addition of CW makes the internal pores of the specimen filled, which makes the chloride ion permeability decrease. The cement hydration products after the addition of FA also make the specimen dense. The addition of CNTs also plays a role in refining the pores to prevent penetration, but because the content of CNTs is less, the effect is smaller. On the contrary, PVA has the least effect on chloride ion penetration, and macrofibres are difficult to have a substantial effect on the microstructure. Cao et al. [59] have shown that the addition of CW can be used as a filler for pores. The addition of CW can refine large pores, and thus the influence index of chloride ion penetration is high. Juliano et al. [60] showed that the minimum value of chloride ion permeability coefficient of the mixture of PVA and steel fibre was greater than 1.0 (10^−12^ m^2^/s). The minimum value of chloride ion permeability coefficient of MSFRCC designed by orthogonal test was 0.88 (10^−12^ m^2^/s), which improved the resistance to chloride ion penetration of cement-matrix composites.

According to Figure 9, when the PVA fibre doping is 1.5%, CW doping is 3%, CNTs doping reaches 0.1%, and FA doping is 40%, the chloride ion permeation coefficient of MSFRCC will reach the minimum, and the optimal ratio of antichloride ion permeation performance is A3B3C1D1 (PVA: 1.5%, CW: 2%, CNTs: 0.1%, FA. 40%).

**Figure 10:**
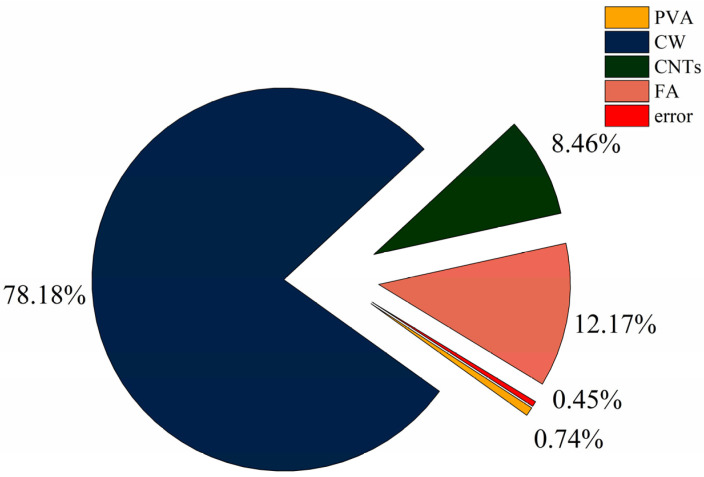
Contribution rate of each influencing factor to chloride ion permeability coefficient.

The results of the ANOVA for chloride ion resistance penetration are shown in Table 12. The distribution of the contributions is shown in Figure 10. According to Table 12, the four factors were ranked in order of significance: B(V_CW_) > D(FA) > C(M_CNTs_) > A(V_PVA_). In Figure 10, CW had the greatest influence on the antichlorine ion permeation performance with a contribution of 78.19%, which was the most important factor affecting the antichlorine ion permeation performance. In addition, CNTs and FA had similar degrees of influence on the antichlorine ion permeation performance with contributions of 8.46% and 12.17%, respectively, which were minor factors affecting the antichlorine ion permeation performance. PVA fibre doping had the least influence on the antichlorine ion permeation performance, with a contribution of only 0.74%.

### 3.5. Grey Correlation Analysis Based on Orthogonal Test Data

The orthogonal test can be used to obtain the optimal ratio scheme for a single evaluation index. However, the orthogonal test cannot satisfy the comprehensive performance optimization scheme for multiple evaluation indices, and the orthogonal test has great limitations for realistic multi-objective problems [61,62]. Grey correlation analysis has been widely used in multi-indicator multi-factor optimization problems [63], which can be used to study the relationship between factors in a system under partially known and partially unknown conditions with small samples and little data. Therefore, in this study, the compressive strength, flexural strength, splitting tensile strength, and chloride ion permeability coefficient were used as evaluation indices. Grey correlation analysis was combined with the entropy weight method to evaluate the comprehensive performance of MSFRCC. The specific steps were as follows:

1. Construction of the reference series A_i_ and the comparison series A_0_.

The reference series A_i_ is constructed with the data of each index, and the comparison series A_0_ is constructed using the optimal value of each evaluation index.

2. Construct the dimensionless reference series Xi and the comparison series X0.

Since each evaluation index has different dimensions and units, to eliminate the influence of data value range and units on the analysis results, the data of each index in the orthogonal group are dimensionless sized. The smaller the value, the better the effect of the cost indicator for the dimensionless processing:

(2) $$x_{ij}=\frac{{\left(a_{j}\right)}_{\max}-a_{ij}}{{{(a}_{j})}_{\max}-{{(a}_{j})}_{\min}}$$

For the benefit metrics with larger values and better effects to be dimensionless:

(3) $$x_{ij}=\frac{a_{ij}-{{(a}_{j})}_{\min}}{{{(a}_{j})}_{\max}-{{(a}_{j})}_{\min}}$$

where Formulas (3) and (4) in (a*_j_*)_max_ is the maximum value of the *j*th index; (a*_j_*)_min_ is the minimum value of the *j*th index; a*_ij_* is the *j*th index value of the *i*th evaluation object, and x*_ij_* is the index value for the dimensionless parameter.

1. Calculating the number of grey correlation coefficients
  (4) $$\xi_{i}(k)=\frac{\underset{i}{\min}\;\underset{k}{\min}\left|x_{0}(k)-x_{i}(k)\right|+\rho \;\underset{i}{\max}\;\underset{k}{\max}\left|x_{0}(k)-x_{i}(k)\right|}{\left|x_{0}(k)-x_{i}(k)\right|+\rho \;\underset{i}{\max}\;\underset{k}{\max}\left|x_{0}(k)-x_{i}(k)\right|}$$
  where the resolution factor in Equation (4) is generally taken as 0.5.

2. Calculate the weights of each evaluation index based on the entropy weighting method.

Construct the index matrix *X_ij_* from the dimensionless evaluation indicators, and calculate the ratio *P_ij_* of the *i*th data under the *j*th indicator to that indicator:

(5) $$P_{ij}=\frac{x_{ij}}{\sum_{i=1}^{n}X_{ij}}$$

Calculate the entropy *e_j_* of the *j*th indicator:

(6) $$e_{j}=-k\sum_{i=1}^{n}P_{ij}In(Pj)$$

(7) $$k=\frac{1}{In(m)}$$

Calculate the information entropy redundancy *d_j_*:

(8) $$d_{j}=1-e_{j}$$

The weights *W_j_* of each indicator are derived from the information entropy redundancy *d_j_*:

(9) $$W_{j}=\frac{d_{j}}{\sum_{j=1}^{m}d_{j}}$$

3. Combining the entropy weight method to calculate the grey correlation of each group of samples:

(10) $$g=\sum_{j=1}^{m}\xi_{ij}w_{j}$$

According to Equation (2) to (10), the grey correlation coefficients and grey correlation degrees of each index for each group of specimens were obtained, as shown in Table 13. The comprehensive performance of the ninth group of specimens was the best, and the grey correlation coefficient reached the maximum value of 0.7915; while the comprehensive performance of the fourth group of specimens was the worst, and the grey correlation coefficient reached the minimum of 0.3794.

**Figure 11:**
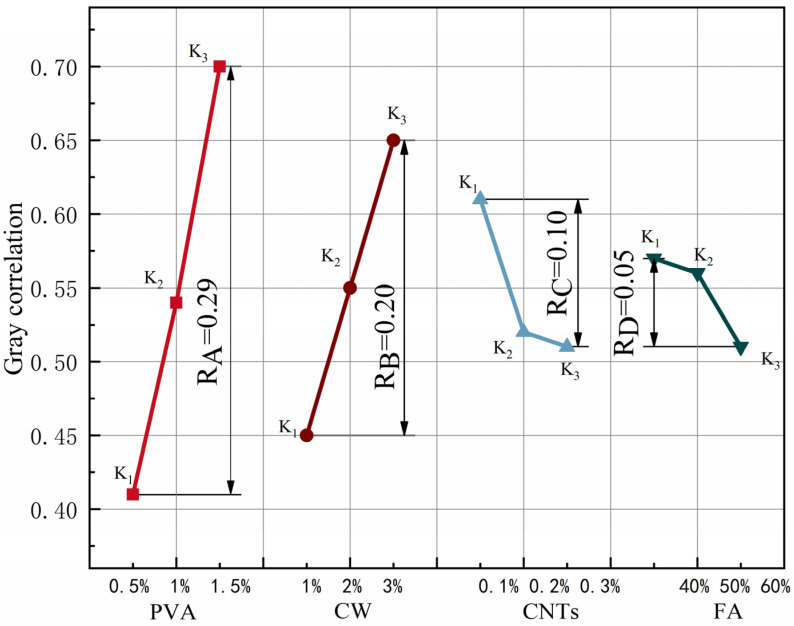
Effect of various factors on the grey correlation degree.

The results of the extreme difference analysis of the grey correlation of each group of specimens are shown in Table 14, and the relationship between different levels of each factor and the correlation is shown in Figure 11. By comparing the R-values between the factors, we can analyse the main order of the influence of the four factors on the grey correlation: R_A_ > R_B_ > R_C_ > R_D_. The effect of PVA fibre on the grey correlation of MSFRCC was the greatest. In the range of PVA fibre doping levels, the grey correlation increased with increasing PVA fibre doping, and when the PVA fibre doping reached 1.5%, the grey correlation was the greatest and the comprehensive performance of the specimens was the best. The effect of CW on flexural strength was second only to that of PVA fibre. The effect of CW doping variation on the grey correlation was similar to that of PVA fibre, and the grey correlation increased with increasing CW doping in the range of doping levels. When the CW doping reached 3%, the grey correlation was the largest, and the comprehensive performance of the specimen was the best. The influence of CNTs and FA on the grey correlation was relatively small, and the influence pattern of these two factors on the grey correlation was opposite to that of PVA fibre and CW. In the range of the doping level, the grey correlation decreased with increasing CNTs and FA doping, and when the CNTs doping reached 0.1% and FA doping reached 40%, the grey correlation was the largest and the comprehensive performance of the specimen was the best. In Table 14, when the PVA fibre dose was 1.5%, the CW dose was 3%, the CNTs dose reached 0.1%, and the FA dose was 40%, the grey correlation of MSFRCC reached the maximum, and the ratio with the best overall performance was A3B3C1D1 (PVA: 1.5%, CW: 2%, CNTs: 0.1%, FA: 40%).

Since the optimized overall optimal fit ratio was not in the orthogonal test design ratio, it was necessary to prepare MSFRCC specimens according to the overall optimal fit ratio and test again; the test results are shown in Table 15. The mean values of the compressive strength, flexural strength, splitting tensile strength, and chloride ion permeability coefficient of the optimized group reached 74 MPa, 12.2 MPa, 5.5 MPa, and 0.83 × 10^−12^ m^2^/s, respectively. Compared with the best data of each index in the orthogonal test, the data of the optimized group showed an 8.9% decrease in compressive strength, a 28.4% increase in flexural strength, a 10% increase in splitting tensile strength, and a 5.7% decrease in chloride ion permeability coefficient. Among the evaluation indices of the optimized group, only the compressive performance was slightly inferior to the optimal group in the orthogonal test, while the flexural performance, splitting tensile performance, and chloride ion penetration resistance were significantly improved compared with the optimal group in the orthogonal test.

The optimal mix ratio of MSFRCC with the best comprehensive performance obtained in this experiment is suitable for the construction of piers for bridges over water and urban pavement in coastal areas. The optimal mix ratio of single performance obtained by orthogonal test design can be applied to different situations for various needs, such as pavement repair or prefabricated plate connection. However. this study lacks durability experiments for MSFRCC in more complex environments. In the future, durability experiments can be performed in different environments.

## 4. Conclusions

In this paper, the effects of different scale fibres on the mechanical properties and chloride ion penetration resistance of cement-based composites were studied. A new type of MSFRCC was designed to improve the multi-scale defects of cement-based composites. The optimal mix ratio of single performance is obtained by orthogonal test design, which is suitable for different MSFRCC mix ratios that can be used under different performance requirements. Finally, the optimal mix ratio of comprehensive performance is obtained by grey correlation analysis. The following are the conclusions of this study:

1. The order of influence on compressive strength was R_B_ > R_C_ > R_A_ > R_D_. CW and CNTs were the main factors that improved the compressive strength, and the contributions of CW and CNTs to the compressive strength were 63.08% and 17.15%, respectively. The optimal mix ratio for compressive properties was A2B3C1D1 (PVA: 1%, CW: 3%, CNTs: 0.1%, FA: 40%).
2. The order of influence on flexural strength was R_A_ > R_B_ > R_D_ > R_C_, and the contribution of PVA fibre to flexural strength was 85.43%. The optimal mix ratio for flexural properties was A3B2C1D2 (PVA: 1.5%, CW: 2%, CNTs: 0.1%, FA: 50%).
3. The order of influence on splitting tensile strength was R_A_ > R_B_ > R_C_ > R_D_, and the contribution of PVA fibre to splitting tensile strength reached 94.72%. The optimal mix ratio for splitting tensile properties was A3B3C1D1 (PVA: 1.5%, CW: 2%, CNTs: 0.1%, FA: 40%).
4. The order of influence on the resistance to chloride ion permeability was R_B_ > R_D_ > R_C_ > R_A_, and the contribution rate of CW to the influence of the chloride ion permeability coefficient reached 78.19%. The optimal mix ratio for chloride ion penetration resistance was A3B3C1D1 (PVA: 1.5%, CW: 2%, CNTs: 0.1%, FA: 40%).
5. The order of the influence of the four factors on the grey correlation degree was R_A_ > R_B_ > R_C_ > R_D_. The optimal mix ratio of comprehensive performance was A3B3C1D1 (PVA: 1.5%, CW: 3%, CNTs: 0.1%, FA: 40%). Compared with the best data of each evaluation index, the compressive strength of the final optimization group decreased by 8.9%, the flexural strength increased by 28.4%, the splitting tensile strength increased by 10%, and the chloride ion permeability coefficient decreased by 5.7%.

## Figures and Tables

**Table 1:** Particle size control index.

Screen size (mm)	2.0	1.6	1.0	0.5	0.61	0.08
Cumulative sieve margin(%)	0	7 ± 4	33 ± 4	67 ± 4	87 ± 4	99 ± 1

**Table 2:** Fibre performance indicators.

PVA Fibre	Diameter (μm)	Length (mm)	Tensile Strength (MPa)	Dry Fracture Tensile (%)	Alkali Resistance (%)
	31	9	1400	17	98
CW	Diameter (μm)	Aspect Ratio	Purity (%)	Specific surface area (m^2^/g)	PH
	1.0	13	98	44.35	7.5
CNTs	Diameter (μm)	Length (mm)	Purity (%)	Specific surface area (m^2^/g)	Ash (wt%)
	8–15	30–50	>95	230	<3

**Table 3:** Factor coding and factor level.

Factors Levels	PVA (A: V_PVA_)	CW (B: V_CW_)	CNTs (C: M_CNTs_)	FA (D: FA)
1	0.5	1	0.1	40
2	1	2	0.2	50
3	1.5	3	0.3	60

**Table 4:** Mix proportion design of MSFRCC.

	Cement (g)	Fly Ash (g)	Sand (g)	Water (g)	PVA (g)	CW (g)	CNTs (g)
1	1620	1080	1350	810	14.6	64.35	2.7
2	1350	1350	1350	810	14.6	128.7	5.4
3	1080	1620	1350	810	14.6	193	8.1
4	1080	1620	1350	810	29.3	64.35	5.4
5	1620	1080	1350	810	29.3	128.7	8.1
6	1350	1350	1350	810	29.3	193	2.7
7	1350	1350	1350	810	43.9	64.35	8.1
8	1080	1620	1350	810	43.9	128.7	2.7
9	1620	1080	1350	810	43.9	193	5.4

**Table 5:** Range analysis of compressive strength.

No.	Ratio Number	Factor/Level				Compressive Strength (MPa)		Average Strength (MPa)	Standard Deviation
		A	B	C	D				
1	A1B1C1D1	1	1	1	1	53.2	52.6	52.9	0.3
2	A1B2C2D2	1	2	2	2	48.5	51.7	50.1	1.6
3	A1B3C3D3	1	3	3	3	58.3	61.3	59.8	1.5
4	A2B1C2D3	2	1	2	3	42.9	46.5	44.7	1.8
5	A2B2C3D1	2	2	3	1	60.9	61.7	61.3	0.4
6	A2B3C1D2	2	3	1	2	83	79.3	81.2	1.85
7	A3B1C3D2	3	1	3	2	47.4	44.7	46.1	1.35
8	A3B2C1D3	3	2	1	3	54.7	57.2	56.0	1.25
9	A3B3C2D1	3	3	2	1	62.8	65.3	64.1	1.25
K1		162.80	143.65	190.00	178.25				
K2		187.15	167.35	158.85	177.30				
K3		166.05	205.00	167.15	160.45				
k1		54.27	47.88	63.33	59.42				
k2		62.38	55.78	52.95	59.10				
k3		55.35	68.33	55.72	53.48				
R		8.12	20.45	10.38	5.93				

**Table 6:** Results of the variance analysis for the compressive strength.

Factor	Sum of Squares	Degree of Freedom	MS	F	P
A	223.04	2.00	116.52	31.45	0.000086809
B	1276.23	2.00	638.11	172.26	0.000000067
C	346.96	2.00	173.48	46.83	0.000017489
D	133.70	2.00	66.85	18.05	0.000708947
Error	33.34	9.00	3.70		

**Table 7:** Range analysis of flexural strength.

No.	Ratio Number	Factor/Level				Flexural Strength (MPa)		Average Strength (MPa)	Standard Deviation
		A	B	C	D				
1	A1B1C1D1	1	1	1	1	5.8	5.6	5.7	0.28
2	A1B2C2D2	1	2	2	2	6.53	6.8	6.7	0.32
3	A1B3C3D3	1	3	3	3	5.6	5.7	5.7	0.25
4	A2B1C2D3	2	1	2	3	6.8	6.2	6.5	0.48
5	A2B2C3D1	2	2	3	1	7.55	8.3	7.9	0.56
6	A2B3C1D2	2	3	1	2	7.5	7.9	7.7	0.38
7	A3B1C3D2	3	1	3	2	9.1	9.4	9.3	0.34
8	A3B2C1D3	3	2	1	3	9.33	9.7	9.5	0.37
9	A3B3C2D1	3	3	2	1	9.2	8.4	8.8	0.58
K1		18.02	21.45	22.92	22.43				
K2		22.13	24.11	21.97	23.62				
K3		27.57	22.15	22.83	21.67				
k1		6.01	7.15	7.64	7.48				
k2		7.38	8.04	7.32	7.87				
k3		9.19	7.38	7.61	7.22				
R		3.18	0.89	0.32	0.65				

**Table 8:** Results of the variance analysis for the flexural strength.

Factor	Sum of Squares	Degree of Freedom	MS	F	P
A	30.60	2.00	15.30	132.88	0.000000208
B	2.52	2.00	1.26	10.96	0.00386743
C	0.37	2.00	0.18	1.59	0.25576187
D	1.29	2.00	0.64	5.59	0.026373986
Error	0.78	9.00	0.12		

**Table 9:** Range analysis of splitting tensile strength.

No.	Ratio Number	Factor/Level				Splitting Tensile Strength (MPa)		Average Strength (MPa)	Standard Deviation
		A	B	C	D				
1	A1B1C1D1	1	1	1	1	2.6	2.5	2.6	0.2
2	A1B2C2D2	1	2	2	2	2.4	2.2	2.3	0.25
3	A1B3C3D3	1	3	3	3	2.5	2.8	2.7	0.3
4	A2B1C2D3	2	1	2	3	3.5	3.3	3.4	0.25
5	A2B2C3D1	2	2	3	1	3.6	3.5	3.6	0.2
6	A2B3C1D2	2	3	1	2	3.7	4	3.9	0.3
7	A3B1C3D2	3	1	3	2	4.5	4.3	4.4	0.25
8	A3B2C1D3	3	2	1	3	4.8	4.7	4.8	0.2
9	A3B3C2D1	3	3	2	1	4.9	5	5.0	0.2
K1		7.50	10.35	11.15	11.05				
K2		10.80	10.60	10.65	10.55				
K3		14.10	11.45	10.60	10.80				
k1		2.50	3.45	3.72	3.68				
k2		3.60	3.53	3.55	3.52				
k3		4.70	3.82	3.53	3.60				
R		2.20	0.37	0.18	0.17				

**Table 10:** Results of the variance analysis for the splitting tensile strength.

Factor	Sum of Squares	Degree of Freedom	MS	F	P
A	14.52	2	7.26	384.35	0.000000002
B	0.44	2	0.22	11.74	0.003107317
C	0.12	2	0.06	3.26	0.085880342
D	0.08	2	0.04	2.21	0.166113385
Error	0.17	9	0.02		

**Table 11:** Range analysis of chloride ion permeability coefficient.

No.	Ratio Number	Factor/Level				Chloride Ion Permeability Coefficient (10^−12^ m^2^/s)		Average (10^−12^ m^2^/s)	Standard Deviation
		A	B	C	D				
1	A1B1C1D1	1	1	1	1	1.89	1.93	1.91	0.17
2	A1B2C2D2	1	2	2	2	1.72	1.73	1.73	0.16
3	A1B3C3D3	1	3	3	3	1.5	1.54	1.52	0.17
4	A2B1C2D3	2	1	2	3	2.83	2.71	2.77	0.21
5	A2B2C3D1	2	2	3	1	1.48	1.62	1.55	0.22
6	A2B3C1D2	2	3	1	2	1.12	1.16	1.14	0.17
7	A3B1C3D2	3	1	3	2	2.68	2.8	2.74	0.21
8	A3B2C1D3	3	2	1	3	1.45	1.49	1.47	0.17
9	A3B3C2D1	3	3	2	1	0.88	0.87	0.88	0.16
K1		5.16	7.42	4.52	4.34				
K2		5.46	4.75	5.37	5.61				
K3		5.09	3.54	5.81	5.76				
k1		1.72	2.47	1.51	1.45				
k2		1.82	1.58	1.79	1.87				
k3		1.70	1.18	1.94	1.92				
R		0.13	1.30	0.43	0.48				

**Table 12:** Results of the variance analysis for the chloride ion permeability coefficient.

Factor	Sum of Squares	Degree of Freedom	MS	F	P
A	0.05	2	0.027	8.67	0.007955105
B	5.27	2	2.635	862.29	0.00000000005
C	0.57	2	0.287	93.83	0.000000939
D	0.82	2	0.408	133.37	0.000000205
Error	0.03	9	0.003		

**Table 13:** Gray correlation coefficient and grey correlation degree.

No.	Gray Correlation Coefficient				Gray Correlation Degree
	Compressive Strength	Flexural Strength	Split Tensile Strength	Chlorine Ion Permeability Coefficient	
1	0.3921	0.3362	0.3557	0.4779	0.3863
2	0.3699	0.4041	0.3333	0.5271	0.4034
3	0.4605	0.3333	0.3655	0.5950	0.4316
4	0.3333	0.3906	0.4609	0.3333	0.3794
5	0.4787	0.5486	0.4862	0.5840	0.5215
6	1.0000	0.5157	0.5464	0.7814	0.7145
7	0.3418	0.8794	0.7067	0.3369	0.5735
8	0.4197	1.0000	0.8689	0.6143	0.7250
9	0.5159	0.7299	1.0000	1.0000	0.7915

**Table 14:** Range analysis of grey correlation degree.

No.	Ratio Number	Factor/Level				Gray Correlation Degree
		A	B	C	D	
1	A1B1C1D1	1	1	1	1	0.3863
2	A1B2C2D2	1	2	2	2	0.4034
3	A1B3C3D3	1	3	3	3	0.4316
4	A2B1C2D3	2	1	2	3	0.3794
5	A2B2C3D1	2	2	3	1	0.5215
6	A2B3C1D2	2	3	1	2	0.7145
7	A3B1C3D2	3	1	3	2	0.5735
8	A3B2C1D3	3	2	1	3	0.7250
9	A3B3C2D1	3	3	2	1	0.7915
K1		1.22	1.34	1.83	1.70	
K2		1.62	1.65	1.57	1.69	
K3		2.09	1.94	1.53	1.54	
k1		0.41	0.45	0.61	0.57	
k2		0.54	0.55	0.52	0.56	
k3		0.70	0.65	0.51	0.51	
R		0.29	0.20	0.10	0.05	

**Table 15:** Performance comparison between optimized MSFRCC and reference MSFRCC.

	Compressive Strength (MPa)	Flexural Strength (MPa)	Splitting Tensile Strength (MPa)	Chloride Ion Permeability Coefficient (10^−12^ m^2^/s)
Reference MSFRCC	81.2	9.5	5.0	0.88
Optimized MSFRCC	74.0	12.2	5.5	0.83
Growth rate (%)	−8.9	28.4	10	−5.7

## Data Availability

The data used to support the findings of this study are available from the corresponding author upon request.
